# Adult *Pgf*^*−/−*^ mice behaviour and neuroanatomy are altered by neonatal treatment with recombinant placental growth factor

**DOI:** 10.1038/s41598-019-45824-6

**Published:** 2019-06-26

**Authors:** Vanessa R. Kay, Lindsay S. Cahill, Anas Hanif, John G. Sled, Peter Carmeliet, Chandrakant Tayade, B. Anne Croy

**Affiliations:** 1 Department of Biomedical and Molecular Sciences, Queen’s University, Kingston, ON K7L 3N6 Canada; 2 Mouse Imaging Centre, Hospital for Sick Children, Toronto, ON M5T 3H7 Canada; 3 Department of Medical Biophysics, University of Toronto, Toronto, ON M5T 3H7 Canada; 4 Laboratory of Angiogenesis and Vascular Metabolism, VIB - Vesalius Research Center, University of Leuven, Department of Oncology, Leuven, Belgium

**Keywords:** Brain, Angiogenesis, Disease model, Development of the nervous system

## Abstract

Offspring of preeclamptic pregnancies have cognitive alterations. Placental growth factor (PGF), is low in preeclampsia; reduced levels may affect brain development. PGF-null mice differ from normal congenic controls in cerebrovasculature, neuroanatomy and behavior. Using brain imaging and behavioral testing, we asked whether developmentally asynchronous (i.e. neonatal) PGF supplementation alters the vascular, neuroanatomic and/or behavioral status of *Pgf*^*−/−*^ mice at adulthood. C57BL/6-*Pgf*^*−/−*^ pups were treated intraperitoneally on postnatal days 1–10 with vehicle or PGF at 10 pg/g, 70 pg/g or 700 pg/g. These mice underwent behavioral testing and perfusion for MRI and analysis of retinal vasculature. A second cohort of vehicle- or PGF-treated mice was perfused for micro-CT imaging. 10 pg/g PGF-treated mice exhibited less locomotor activity and greater anxiety-like behavior relative to vehicle-treated mice. Depressive-like behavior showed a sex-specific, dose-dependent decrease and was lowest in 700 pg/g PGF-treated females relative to vehicle-treated females. Spatial learning did not differ. MRI revealed smaller volume of three structures in the 10 pg/g group, larger volume of seven structures in the 70 pg/g group and smaller volume of one structure in the 700 pg/g group. No cerebral or retinal vascular differences were detected. Overall, neonatal PGF replacement altered behavior and neuroanatomy of adult *Pgf*^*−/−*^ mice.

## Introduction

Serial measurements of maternal plasma across gestation have strongly linked altered pro- and anti-angiogenic signals to the pathogenesis of preeclampsia (PE). Conceptus-derived placental tissue is considered the major source of these molecular aberrations with delivery of the placenta alleviating the syndrome in most patients. Placental growth factor (PGF), a pro-angiogenic, vascular endothelial growth factor (VEGF)-related molecule, is inappropriately low in maternal circulation well in advance of clinical presentation in many preeclamptic gestations^1–3^. PGF production may be low in developing fetuses as well as in their placentas since cord blood and amniotic fluid PGF concentrations correlate with maternal blood concentrations in healthy pregnancies^3,4^. Lower neonatal cord blood PGF concentrations have also been reported after a PE gestation^5^. PGF is expressed by conceptuses at all pre- and post-implantation stages of pregnancy in humans, mice and other species^6–8^. In healthy human pregnancies, maternal PGF concentrations are linked to fetal growth^9–11^ and adolescent retinal arterial diameter^12^. Therefore, low fetal PGF concentrations during PE may impair fetal development, including development of brain and the brain’s vascular components.

Offspring of preeclamptic pregnancies (PE-F1s) demonstrate lower scores on cognitive testing as children, young adults or older adults^13–17^. These individuals also have a higher risk of neuropsychiatric disorders including attention deficit/hyperactivity disorder^18^, autism spectrum disorder^19^ and depression^20^. Cognitive alterations are postulated to relate to underlying PE-induced changes to brain structure and vasculature during vulnerable periods in development. A small pilot study of 7–10 year old PE-F1s documented larger relative volumes of the brainstem, cerebellum, amygdala and temporal lobe^21^. These children also had significantly smaller vessel diameters in the occipital and parietal lobes^21^. Analyses of the Helsinki birth cohort identified greater stroke risk in adult PE-F1s that was postulated to be due to alterations in the cerebrovasculature arising during developmental insult^22^, perhaps stemming from a deficit in production of angiogenic factors including PGF.

In stark contrast to the critical roles of VEGF and its signalling via VEGF receptor 2 (VEGFR2) during mouse development, PGF gene deletion is compatible with life. Although viable, *Pgf*^*−/−*^ mice are well-known to demonstrate impairments of angiogenesis in ischemic and inflammatory contexts^23^. PGF works through VEGF receptor 1 (VEGFR1) and neuropilin-1. Other VEGF family members and semaphorin3A work through these same receptors suggesting when PGF is absent, other ligands sustain receptor function^23^. PGF’s important roles have been identified in collaterogenesis, arteriogenesis and wound healing^23^. Recent work also identified delayed hindbrain vascularization during development in *Pgf*^*−/−*^ mice^24^ and distorted organization of the arteries and veins in vascularizing neonatal and mature adult *Pgf*^*−/−*^ retinas^25^. In *Pgf*^*−/−*^ mice, altered fetal and adult circle of Willis connectivity is apparent and appears to increase vulnerability to stroke^24^. Robust analysis of the cerebrovasculature using micro-computed tomography (μCT) imaging found a greater number of small-diameter vessels in the brains of *Pgf*^*−/−*^ mice relative to wild-type control^26^. Magnetic resonance imaging (MRI) showed associated alterations in neuroanatomy with smaller total volume of the brain and smaller volume of 10 of 62 measured discrete structures^26^. *Pgf*^*−/−*^ mice also exhibited behavioral alterations with less depressive-like behavior on the tail suspension test (TST), less object exploration on the serial dishabituation test, better object discrimination on the novel object recognition test and poorer spatial memory on the y-maze spontaneous alternation test (YMSAT)^26^. *Pgf*^*−/−*^ mice can therefore model the effects of a low PGF PE-gestation on offspring brain and cognition. A recent study reported impaired brain vascularization in mice after tissue-restricted knockdown of PGF expression in the placenta^27^. As such, deficits in both local brain-produced PGF and circulating placenta-produced PGF may impair brain vascularization and neuroanatomy. Replacement of PGF could ameliorate or even reverse these changes.

The safety and efficacy of PGF therapy have been investigated in models of ischemic stroke and myocardial infarction. In ischemic myocardial injury, PGF treatment or transduction improved cardiac performance and increased vascularization including vessel number and perfusion^28–30^ as well as vessel maturation and macrophage recruitment^31^. Treatment with PGF was also protective and angiogenic after an isoprenaline challenge in rabbits^32^. In rodent models of stroke, transfer of mesenchymal and monocyte-derived multipotential cells engineered to express PGF decreased infarct size while increasing angiogenesis^33,34^. Comparison of angiogenic factors in the brain revealed PGF as the most promising angiogenic factor for promotion of angiogenesis without causing excessive vessel permeability, edema, inflammation or scarring^35^. However, *in utero* replacement is complicated by the consistent expression of PGF during brain development and by the placenta’s barrier function which may prevent maternally-administered PGF, a large 25 kDa molecule, from reaching the fetus. Since the neonatal brain is plastic, we questioned if a therapeutic window was present in early life during which angiokine replacement could alter the neuroanatomy and cerebrovasculature in *Pgf*^*–/-*^ mouse brains and change adult behavior. Here *Pgf*^*−/−*^ mice were treated from postnatal day (P)1 to P10 with PGF at doses corresponding to the mouse non-pregnant adult and peak-pregnancy circulating concentrations as well as a supraphysiological dose^36^. As adults, the cerebrovasculature, neuroanatomy and behavior of the treated mice were assessed.

## Methods

### Animal use

All protocols using animals were reviewed and approved by the Queen’s Animal Care Committee and were in accordance with the national recommendations for the ethical use of animals in research. A *Pgf*^*−/−*^ mouse colony originating from mice provided by Dr. P. Carmeliet (Vesalius Research Centre, Leuven, Belgium) was maintained at Queen’s University using *Pgf*^*−/−*^ by *Pgf*^*−/−*^ breeding pairs in ventilated microisolator cages with barrier husbandry and a 12 h light and 12 h dark cycle. *Pgf*^*−/−*^ pups were weighed and treated daily from P1 to P10 with intraperitoneal (i.p.) injections of recombinant PGF (465-PL/CF, R & D Systems, Minneapolis, MN, USA) or phosphate buffered saline (PBS; 10010023, Life Technologies, ThermoFisher Scientific, Burlington, ON, Canada) as a control. Three doses of PGF (10 pg/g, 70 pg/g and 700 pg/g) were used to mimic the mouse non-pregnant adult circulating concentrations, maternal peak pregnancy concentrations and supraphysiological levels of PGF respectively^36^. The volume of the injection given was based on weight with the PGF treatments diluted so that the same volume could be given for each dose level. The same weight-based volume of PBS was given to control pups. 8 litters (n = 51 pups) were treated with PBS, 11 litters (n = 75 pups) were treated with 10 pg/g PGF, 6 litters (44 pups) were treated with 70 pg/g PGF and 4 litters (n = 37 pups) were treated with 700 pg/g PGF. Additional litters were treated until at least 16 male and 16 female mice per treatment group were obtained. The male:female sex ratio was 1.45 when calculated from 159 pups. Treated pups were aged to adulthood for behavioral testing (n numbers reported in figure legends as equipment malfunctions resulted in variability in n number between tests). Sixteen mice (8 males and 8 females) from each treatment group were randomly selected and prepared for MRI at 5–6 months of age. Eyes were collected from perfused animals for whole mount immunofluorescence analysis of the retinal vasculature (n = 10 mice per group). An additional randomly selected 16 mice (8 male and 8 females) from each treatment group were prepared for µCT imaging at 5–6 months of age.

### Behavioral testing

Treated mice aged 4–5 months underwent three behavioral tests in the order described below. These tests were selected from previous studies in which they were found to identify differences between *Pgf*^*−/−*^ and *Pgf*^*+/+*^ mice^26^. Each mouse experienced each behavioral test once. All tests were conducted in the same dedicated, dimly-lit room during the dark period. Mice were transferred to the behavioral testing room during the light period one hour before the dark period for acclimatization.

#### Open field test

To assess locomotor activity and anxiety-like behavior, treated mice performed the open field test (OFT). Mice were individually placed in a 45 cm × 45 cm arena with Actimot infrared sensors (SPE Limited) and allowed to explore freely for 10 min. Time moving, total distance travelled, number of times rearing and the percent of time spent in the center were recorded by the computer.

#### Tail suspension test

Depressive-like behavior was examined using the TST. Mice were suspended by their tails for a 6 min period with a plastic cover over the tail to prevent tail climbing^37,38^. The time until the first episode of immobility (latency to immobility), the total time immobile and the total number of immobile episodes were quantified. Immobility was defined as the mouse hanging passively with no purposeful movements of the body or legs.

#### Y-maze spontaneous alternation test

Spatial learning was examined using the YMSAT. Mice were placed in a maze with three arms and allowed to freely explore. As mice should explore each arm sequentially, the YMSAT was scored by calculating the number of correct triads where the mouse enters the three unique arms consecutively as a percentage of total number of entries (percent alternation = correct triads/(total entries − 2) * 100).

### Magnetic resonance imaging assessment of neuroanatomy

#### Gadolinium contrast perfusion and fixation

Adult treated mice (5–6 months) that had undergone behavioral testing were perfused with fixative and gadolinium contrast for MRI assessment of neuroanatomy as described by Cahill *et al*.^39^. Eight mice of each sex were perfused and imaged for each treatment group. Mice were anesthetized with an overdose of sodium pentobarbital (75 mg/kg, i.p) and perfused through the left ventricle with 30 mL PBS containing 10U/ml heparin and 2 mM gadolinium contrast (ProHance, Bracco Diagnostics Inc., Princeton, NJ) at a rate of 1 mL/minute. Next, the mice were fixed with a 30 mL perfusion of 4% paraformaldehyde (PFA, Electron Microscopy Services) with 2 mM gadolinium contrast. Extracranial soft tissue was removed from the isolated heads which were post-fixed in 4% PFA overnight and then stored in PBS with gadolinium contrast and 0.01% sodium azide (SAZ001, Bioshop) until imaging. Imaging was completed at least 28 days after fixation to allow for uniform tissue swelling between the samples^40^.

#### Magnetic resonance imaging and analysis

A multi-channel 7.0 Tesla MRI scanner (Agilent Inc., Palo Alto, CA, USA) was used to image the brain. Sixteen custom-built solenoid coils were used to image 16 brains simultaneously^41,42^. Anatomical MRI scans were completed using a T2-weighted, 3D fast spin-echo sequence using a cylindrical *k*-space acquisition, with a TR of 350 ms and TEs of 12 ms per echo for 6 echoes, four averages, field-of-view of 20 mm × 20 mm × 25 mm and matrix size = 504 × 504 × 630 giving an image with 0.040 mm isotropic voxels^43^. To compare changes in the mouse brain, the images were linearly (6 parameter followed by a 12 parameter) and non-linearly registered together using an automated image registration approach implemented in the Pydpiper toolkit^44^ to create an average image of all the scans^45^. The deformations needed to take each individual mouse’s anatomy into this final average space were analyzed^46,47^. The Jacobian determinants of the deformation fields were then calculated as an estimate of the local volume changes at each voxel for comparison between the groups. Using the results of the linear alignment, multiple templates of a segmented anatomical atlas with 62 labelled structures^48^ including the cortical lobes, corpus callosum, ventricles, cerebellum, brain stem, and olfactory bulbs were created (the MAGeT procedure)^49^. From the final voted segmentation, absolute (mm^3^) structure volumes were calculated for each image.

### Micro-computed tomography assessment of cerebrovasculature

#### Microfil perfusion and fixation

Sixteen mice from each treatment group (n = 8 males and 8 females per group) at age 5–6 months were perfused with Microfil contrast (MV-122 Microfil; Flow Technology, Carver, MA) using a Servo pressure pump (PS-200-P, Living Systems Instrumentation) according to the protocol reported by Ghanavati *et al*.^50^. for μCT imaging. Briefly, mice were anesthetized with an overdose of sodium pentobarbital (75 mg/kg i.p) and injected with 200 U heparin. The inferior vena cava was tied off and the thoracic aorta was clamped to direct the perfused reagents to the cerebrovasculature. Mice were first perfused through the left ventricle with PBS containing 5 U/mL heparin at 50 mmHg of pressure. The carotid arteries were clamped for a 2 min perfusion with Microfil under 150 mmHg of pressure. Then the carotids were unclamped for a further 20 min perfusion with Microfil under 150 mmHg of pressure. After perfusion, the Microfil polymerized under 30 mmHg of pressure for 90 min. The decapitated heads were dissected. The isolated skulls were fixed overnight in 10% formalin then decalcified in 8% formic acid (F0507, Sigma-Aldrich) for 48 hours. Finally, the skulls were rinsed and embedded in 1% agarose gel (AGA001–100, Bioshop) for storage and imaging.

#### μCT imaging and analysis

Images were acquired using a Bruker Skyscan 1272 micro-CT scanner (Bruker Skyscan, Belgium). The brains were scanning using an X-ray source at 80 kV and 125 μA. The specimen was rotated 180° in 0.2° increments, generating 900 views which were reconstructed into data blocks with a 12 μm voxel size. Voxel intensities in the reconstructed images were normalized to be between 0 and 1. The scans were registered to a common space as previously described^51^ and manually segmented to exclude vessels outside the brain using Display software (Montreal Neurological Institute, Canada). The structure of the vasculature was identified automatically using a previously described algorithm^52^. The contrast parameter in the vessel tracking algorithm, which describes the difference in intensity between voxels that is required to differentiate vessel from background, was set to either 0.4 or 0.3 to optimize tracking of the vessels visible in the scan. Vessel segments with diameter less than 0.04 mm were excluded due to low imaging reliability for vessels this size. Total vessel segment numbers and diameters were extracted for comparison between groups.

### Retinal vasculature whole mount immunofluorescence

Eyes were collected from 10 mice from each treatment group (n = 5 male and 5 female except in the 70 pg/g group where n = 4 male and 6 female) that were perfused for MRI. The fixed eyes were dissected to isolate the retina. The retinal vessels were identified using Alexa Fluor 594-conjugated *Griffonia simplicifolia* isolectin B4 (IB4, Life Technologies). Vascular smooth muscle was identified on retinal arteries with Alexa Fluor 488-conjugated anti-actin (53-6496-82, eBioscience, San Diego, CA, USA). Briefly, retinas were incubated in 0.2% bovine serum albumin and 0.5% Triton-X (TRX506.500, Bioshop) for blocking, washed in 1% Triton-X in PBS and stained overnight with IB4 and anti-actin. The retinal vasculature was visualized at 25X, 50X, 100X, 200X and 400X using an M1 Imager microscope with Axiovision (Zeiss, Toronto, ON). Vessel parameters were assessed by a blinded investigator using ImageJ (National Institutes of Health, Bethesda, MD) and Angiotool^53^.

### Statistical analysis

Pup weights over the 10 days of treatment were compared using a linear model including day and treatment as independent variables. A linear mixed effects model that incorporated litter as an independent variable was also used to correct for litter-related confounders like litter size. Adult weights in the treatment groups were compared using a one-way ANOVA to compare treatment groups and a two-way ANOVA to compare treatment groups and sex. Performance on the cognitive behavioral tests was analyzed using one-way ANOVAs or Kruskal-Wallis tests for the aggregate treatment groups and two-way ANOVAs for sex-specific analysis. Volume of brain structures was assessed using a False Discovery Rate of 0.1 to correct for multiple comparisons. Vessel segment number was examined using cumulative frequency histograms of vessel segment number with diameter. Spline models were fitted to the histograms with knots at 100, 200, 300 and 400 μm. The spline coefficients were compared between groups using a linear mixed effects model that incorporated treatment group, coefficient and contrast at which the vessel-tracking algorithm was run. Sex was added to the model for a sub-analysis with sex-stratification. A sensitivity analysis in which all samples run at a contrast of 0.4 were excluded was also completed. Retinal vascular parameters were assessed using one-way ANOVA to compare treatment groups and two-way ANOVA to compare treatment group stratified by sex.

## Results

### Weights

**Figure 1:**
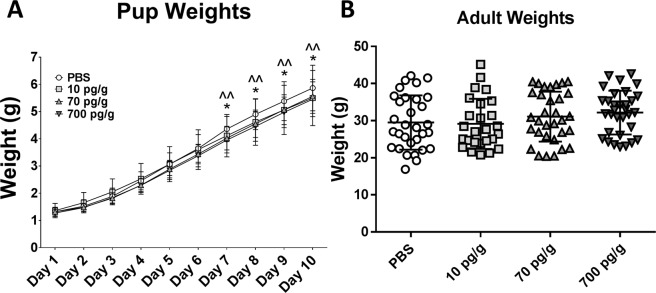
Comparison of pup weights from P1–10 (**A**) showed that 70 pg/g (significance demonstrated by *) and 700 pg/g (significance demonstrated by ^) PGF-treated pups weighed significantly less than PBS-treated pups beginning on day 7. There was no significant difference between 10 pg/g PGF-treated and PBS-treated pup weights. There was also no significant difference between the 10 pg/g, 70 pg/g and 700 pg/g PGF-treated pup weights at any time point. At euthanasia and perfusion, there was no difference in adult weight between any of the treated groups (**B**). Numbers analyzed were n = 33 treated with PBS (average age 16 weeks), n = 27 treated with 10 pg/g PGF (average age 20 weeks), n = 33 treated with 70 pg/g (average age 18 weeks), and n = 32 treated with 700 pg/g PGF (average age = 20 weeks). Graphs show mean ± SD. * corresponds to p < 0.05, ^^ corresponds to p < 0.01.

Pups were weighed daily from P1 to P10 (Fig. 1A). From day 7 onward, 70 pg/g and 700 pg/g PGF-treated pups weighed significantly less than PBS-treated pups (p < 0.05 and p < 0.01, respectively). 10 pg/g PGF-treated pups and PBS-treated pups had similar weights at all time points. After statistical correction for litter using a linear mixed effect model with litter as a random effect, there was no significant difference between the PGF- and PBS-treated groups on any day. Two pups in the PBS control group, six pups in the 10 pg/g PGF-treated group and one pup in the 70 pg/g PGF-treated group died before P10. No pups died in the 700 pg/g PGF-treated group. There was no significant difference in adult body weight between any of the groups (Fig. 1B). When males and females were analyzed separately, there was no difference between treatment groups although, as expected, males were significantly heavier than females (data not shown).

### Behavioral testing

#### Open field test

**Figure 2:**
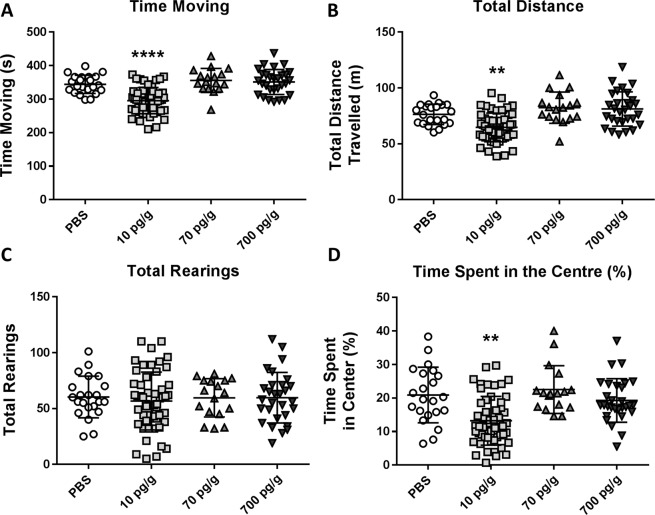
In the OFT, 10 pg/g PGF-treated mice spent less time moving compared to the other groups (**A**) and travelled less distance in total compared to the other groups (**B**) although there was no difference between the groups with respect to number of times rearing (**C**). 10 pg/g PGF-treated mice also spent a lower percentage of time in the centre compared to the other groups (**D**). Graphs show individual values with mean ± SD. ** corresponds to p < 0.01 compared to the PBS-treated group. **** corresponds to p < 0.0001 compared to the PBS-treated group. Significance between the 10 pg/g and 70 pg/g or 700 pg/g groups is not shown. Numbers analyzed were n = 22 treated with PBS, 53 treated with 10 pg/g PGF, 17 treated with 70 pg/g PGF and 29 treated with 700 pg/g PGF.

In comparisons between *Pgf*^*−/−*^ mice treated with PBS or PGF groups, 10 pg/g PGF-treated mice spent less time moving (Fig. 2A) and travelled less distance overall (Fig. 2B) compared to the other treatment groups, suggesting decreased locomotor activity at this dose. However, the 10 pg/g PGF-treated mice did not exhibit a difference in the number of times rearing. The 10 pg/g PGF-treated mice also spent significantly less of the time in the center of the arena than the PBS, 70 pg/g and 700 pg/g PGF-treated mice (Fig. 2D) suggesting an increase in anxiety-like behavior. Similar results were seen after sex stratification for both males and females (data not shown). Time moving was significantly decreased in both male and female 10 pg/g PGF-treated mice (p = 0.0094 and p = 0.0010 respectively). Total distance travelled was significantly decreased in female (p = 0.0311) and non-significantly decreased in male 10 pg/g PGF-treated mice (p = 0.2516 respectively). There was no difference in the number of rearings between groups in males or females. Finally, male 10 pg/g PGF-treated mice showed a significant decrease in percent of time spent in the center (p = 0.0202) while female 10 pg/g PGF-treated mice exhibited a non-significant decrease in percent of time spent in the center (p = 0.2777).

#### Tail suspension test

**Figure 3:**
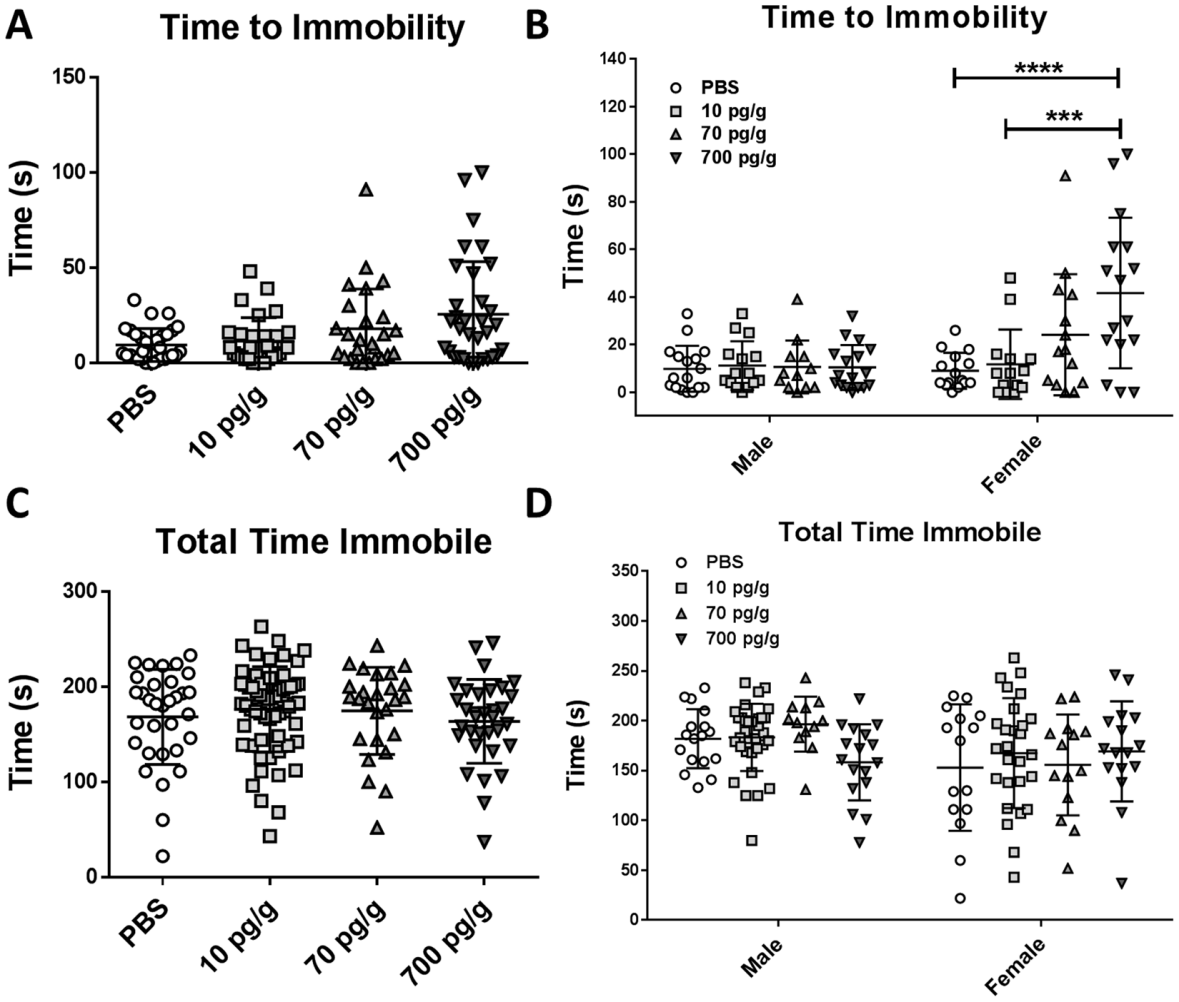
In the tail suspension test, there was a trend for 700 pg/g treated mice to demonstrate longer times to immobility than PBS- and 10 pg/g PGF-treated mice (**A**). After sex stratification, female but not male 700 pg/g PGF-treated mice had a significantly longer time to immobility than PBS- or 10 pg/g PGF-treated mice (**B**). There was no difference in total time immobile between any of the groups (**C**). After sex stratification, all the groups had similar total times immobile (**D**). Graphs show individual values with mean ± SD. Numbers analyzed were n = 31 treated with PBS, 30 treated with 10 pg/g PGF, 26 treated with 70 pg/g PGF and 33 treated with 700 pg/g PGF for time to immobility and n = 31 treated with PBS, 62 treated with 10 pg/g PGF, 26 treated with 70 pg/g PGF and 33 treated with 700 pg/g PGF for total time immobile.

PBS and PGF-treated *Pgf*^*−/−*^ mice had statistically similar times to immobility in the TST (Fig. 3A). After sex-stratification, 700 pg/g PGF-treated female mice exhibited significantly greater time to immobility compared to PBS and 10 pg/g PGF-treatment (Fig. 3B). However, total time spent immobile did not differ between any of the groups (Fig. 3C) even after sex stratification (Fig. 3D).

#### Y-maze spontaneous alternation test

**Figure 4:**
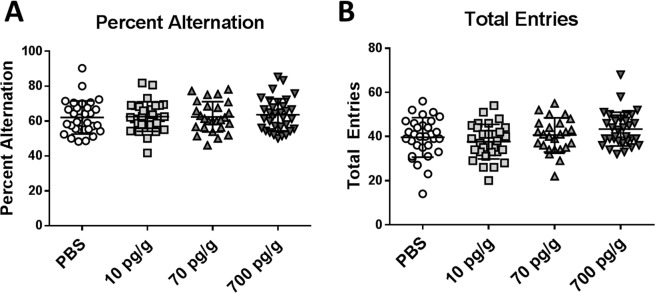
In the Y-maze spontaneous alternation test, all groups performed similarly and achieved similar percent alternation scores (**A**). No difference was identified in the total number of arm entries (**B**). Graphs show individual values with mean ± SD. Numbers analyzed were n = 31 treated with PBS, 30 treated with 10 pg/g PGF, 26 treated with 70 pg/g PGF and 33 treated with 700 pg/g PGF.

All PBS- and PGF-treated *Pgf*^*−/−*^ mice performed similarly on the YMSAT test (Fig. 4A) suggesting similar spatial learning and no effect of treatment. There was no difference in the total number of entries made in any group (Fig. 4B). Sex-stratification did not reveal a sex-specific effect of treatment on performance (data not shown).

### Altered brain structure volume in adult postnatally-treated mice

**Figure 5:**
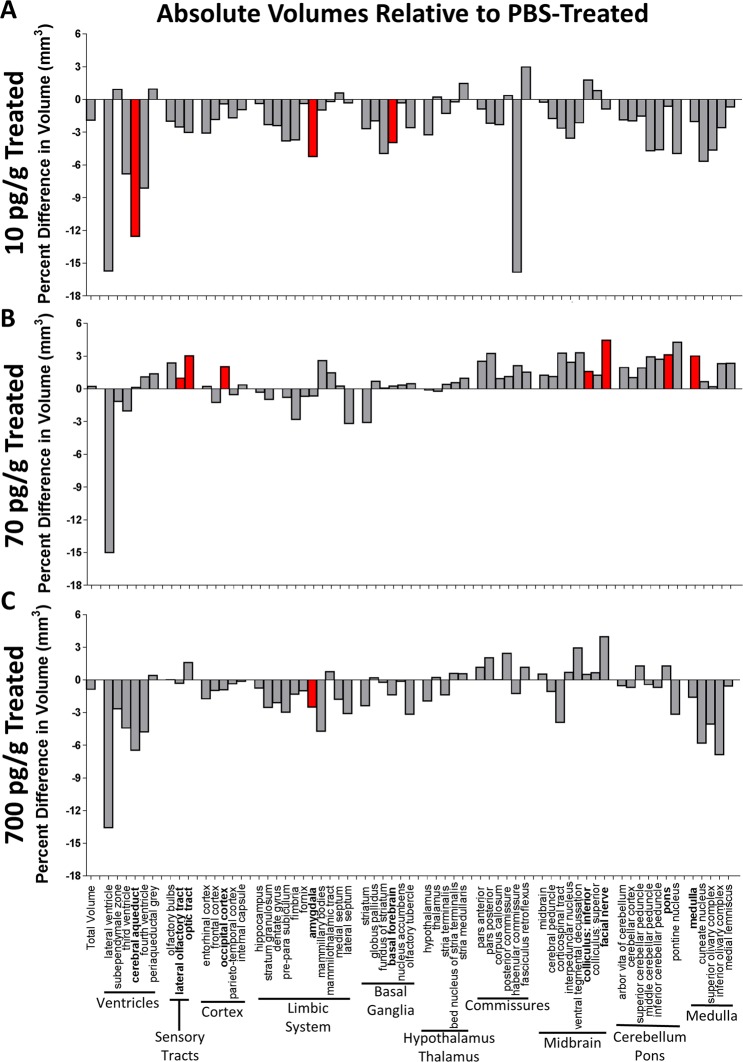
MRI-assessed absolute volume of 62 brain structures relative to PBS-treated controls. The percent difference in absolute volume of 62 brain structures compared to PBS-treated control was determined for 10 pg/g (**A**), 70 pg/g (**B**) and 700 pg/g (**C**) PGF-treated mice. There were 16 mice per treatment group with 4 excluded for poor perfusion quality. Significance was tested using a FDR of 0.1 and is presented in red.

Sixteen mice from each treatment group were perfused for MRI volumetric analysis. 1 PBS-treated male, 1 of the 10 pg/g PGF-treated females and 2 of the 10 pg/g PGF-treated males were excluded for poor perfusion quality. Absolute volume of 62 brain structures was assessed and presented as percent differences in absolute volume relative to PBS-treated mice (Fig. 5). Overall, treatment groups tended to have smaller ventricular volumes, with significantly smaller cerebral aqueduct volume in the 10 pg/g PGF-treated group. Surprisingly, despite the decrease in ventricular volume, there was no difference in total brain volume. The 70 pg/g PGF-treated group had significantly larger lateral olfactory and optic tracts, although these structures were non-significantly smaller in the 10 pg/g and 700 pg/g PGF-treated groups. Only the 70 pg/g PGF-treated group had significantly different volume of the cortex with greater occipital lobe cortex volume. The limbic system structure volumes tended to be non-significantly smaller in all treatment groups, with significantly smaller amygdala volume in 10 pg/g and 700 pg/g PGF-treated groups. The 10 pg/g PGF-treated group also tended to have smaller basal ganglia volumes, with a significant difference in basal forebrain volume. However, there was no clear trend for volume change in the basal ganglia at higher doses. There was also no clear change in hypothalamic/thalamic or commissure volumes in any of the treatment groups. Finally, brainstem and cerebellar structure volumes tended to be greater in the 70 pg/g PGF-treated group, with significantly larger inferior colliculus and facial nerve, as well as larger pons and medulla. Conversely, the 10 pg/g and 700 pg/g PGF-treated groups had non-significantly smaller brainstem and cerebellar structures. In general, the 70 pg/g PGF-treated group tended to have larger brain structure volumes while 10 pg/g and 700 pg/g PGF-treated mice tended to have smaller brain structure volumes relative to PBS-treated control. The relative volume differences (data not shown) were similar to the absolute volumes, suggesting an isolated effect on the impacted structures.

When absolute volumes of the brain structures that were significantly different were compared, a linear dose response was not seen (Supplementary Fig. 1A–J). In the lateral olfactory tract, optic tract, occipital lobe cortex, facial nerve, inferior colliculus, pons and medulla, structure volume was significantly greater in the 70 pg/g PGF-treated group but was similar to PBS-treated control at the 10 pg/g and 700 pg/g doses. Conversely, the cerebral aqueduct and basal forebrain were smaller in the 10 pg/g PGF-treated group and the amygdala was smaller in the 10 pg/g and 700 pg/g PGF-treated groups compared to PBS-treated control. Significant sex*dose interactions were identifiable for the volumes of 5 structures (Supplementary Fig. 1K–O). Lateral olfactory tract volume shrunk with increasing dose in males but appeared to be larger in volume at the 70 pg/g dose in females (Supplementary Fig. 1K). Likewise, the inferior colliculus volumes were larger at higher doses in males and smaller at higher doses in females (Supplementary Fig. 1L). Conversely, the volumes of the amygdala (Supplementary Fig. 1M), nucleus accumbens (Supplementary Fig. 1N) and arbor vitae of cerebellum (Supplementary Fig. 1O) tended to be larger at high doses in males and smaller at high doses in females.

**Figure 6:**
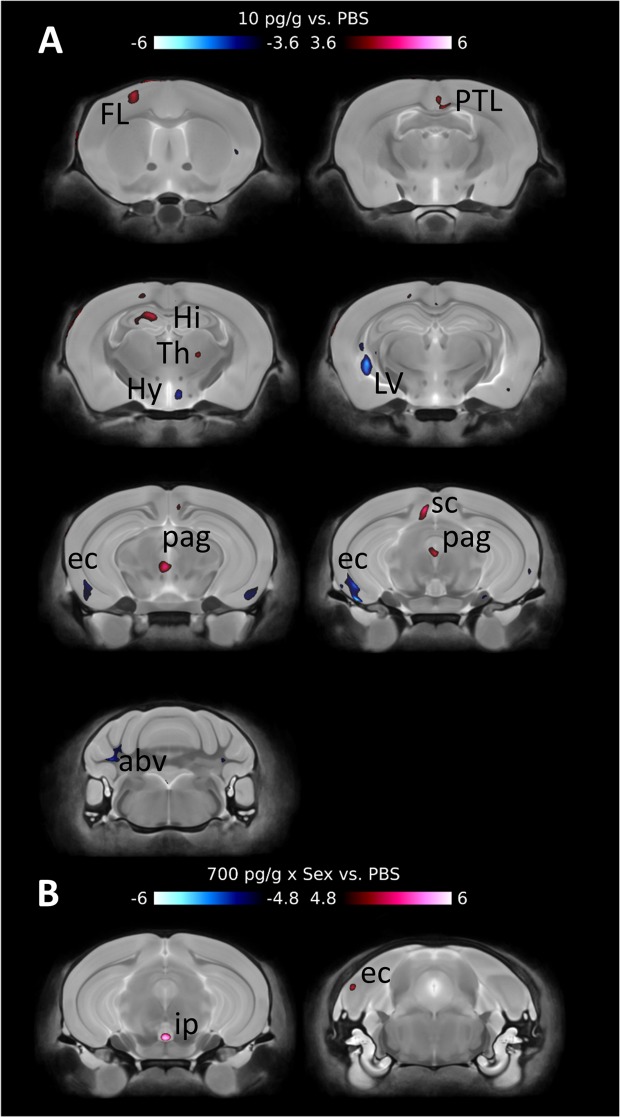
Voxel-wise comparison revealed minor differences between the PBS- and 10 pg/g PGF-treated mice (**A**). When sex*dose interactions were examined, sex was only found to be important in the comparison between PBS- and 700 pg/g PGF-treated mice (**B**). Red corresponds to significantly larger areas. Blue corresponds to significantly smaller areas. There were 16 mice per treatment group with 4 excluded for poor perfusion quality. Structures are identified as abv: arbor vita of cerebellum; EC: entorhinal cortex; FL: frontal lobe; Hi: hippocampus; Hy: hypothalamus; ip: interpedunclar nucleus; LV: lateral ventricle; pag: periaqueductal grey matter; PTL: parietotemporal lobe; sc: superior colliculus; Th: thalamus. Data were analyzed using a FDR of 0.1 for multiple comparisons.

Voxel-wise comparison of brain volume identified few differences (Fig. 6). There were small areas of significantly greater volume (red) in the frontal lobe, hippocampus, thalamus and periaqueductal grey matter in the 10 pg/g PGF-treated mice. There were also small areas of significantly smaller volume (blue) in the hypothalamus, lateral ventricle, entorhinal cortex and arbor vitae in the cerebellum. Voxel-wise comparison also identified areas in the interpeduncular nucleus and entorhinal cortex that exhibited a dose*sex interaction in the 700 pg/g PGF-treated mice.

### µCT-assessed cerebrovasculature in adult postnatally-treated mice

**Figure 7:**
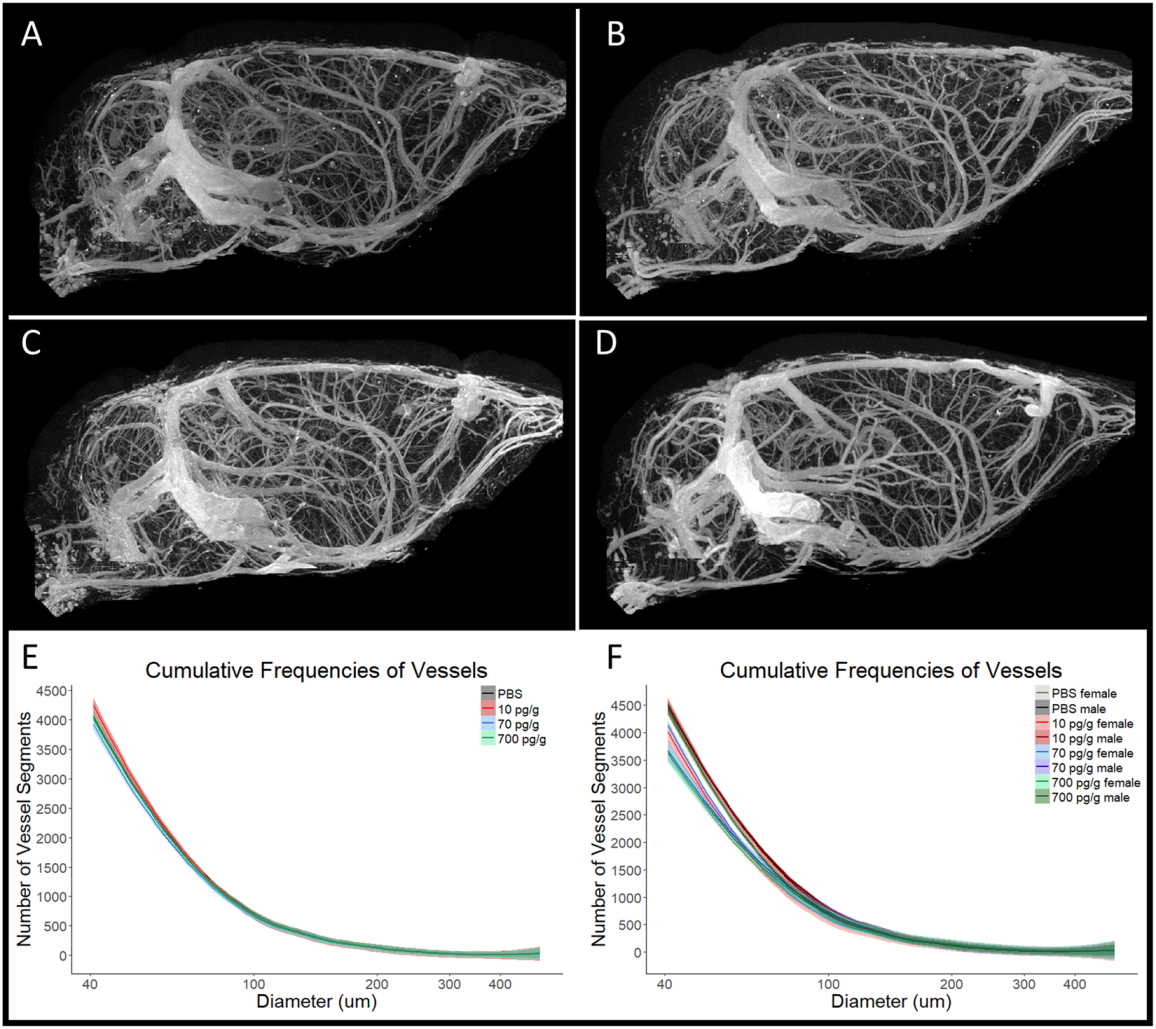
µCT imaging was used to examine the number and diameter of vessels in the brain. Representative images are shown of PBS-treated (**A**), 10 pg/g PGF-treated (**B**), 70 pg/g PGF-treated (**C**) and 700 pg/g PGF-treated (**D**) mouse brain. There was no significant difference in the cumulative frequencies of vessel segments between treatment groups (**E**). There was also no significant difference between treatment groups when males and females were analyzed separately (**F**). Cumulative frequency histograms were analyzed using fitted spline models and linear modelling of the spline coefficients. Graphs show averaged cumulative frequency histograms with 95% confidence interval. In the cumulative frequency histograms, PBS-treated mice are represented in grey, 10 pg/g PGF-treated mice in red, 70 pg/g PGF-treated mice in blue and 700 pg/g PGF-treated mice in green. When separated by sex, females are represented by lighter shades. There were 16 mice per treatment group with 1 excluded for failure of the vessel-tracking algorithm.

Sixteen mice per treatment group were prepared for µCT imaging of the cerebrovasculature. One 70 pg/g PGF-treated male was excluded due to non-completion of the vessel-tracking algorithm. Representative 2D images of the 3D µCT scans of the cerebrovasculature are shown in Fig. 7A–D. Vessel segment number by diameter was examined using a linear model to compare the spline coefficients of the cumulative frequencies. Treatment group was not significant in the model (F = 0.759, p = 0.518) and there was no significant difference between any of the groups with post-hoc comparisons (Fig. 7E). Sex but not the sex*treatment interaction was significant (F = 8.656, p = 0.003 and F = 2.398, p = 0.068, respectively). There were no significant differences between groups on post-hoc testing when stratified by sex (Fig. 7F). Overall, early postnatal treatment with PGF did not impact vessel segment number or diameter in the adult mouse.

### Retinal vasculature in treated mice

The retinal vasculature was assessed in male and female mice (n = 10 mice per treatment group) from each treatment group after perfusion for MRI. Leaked red blood cells from the perfusion obscured vessels in some retinas, preventing some parameters from being evaluated. The number of crossovers and abnormal veins could not be evaluated in three of the 10 pg/g PGF-treated males and one of the 70 pg/g PGF-treated males. Distance from the optic disc to the plexus could not be evaluated in two of the 70 pg/g PGF-treated mice and two of the 700 pg/g PGF-treated mice. Arterial diameter and venous diameter could not be evaluated in one of the 70 pg/g treated females and one of the 700 pg/g PGF-treated males. Angiotool measurements including vessel percentage of area, junction density, total and average vessel length and number of end points could not be evaluated in one of the 10 pg/g PGF-treated males. All other measurements included all ten animals from each treatment group.

**Figure 8:**
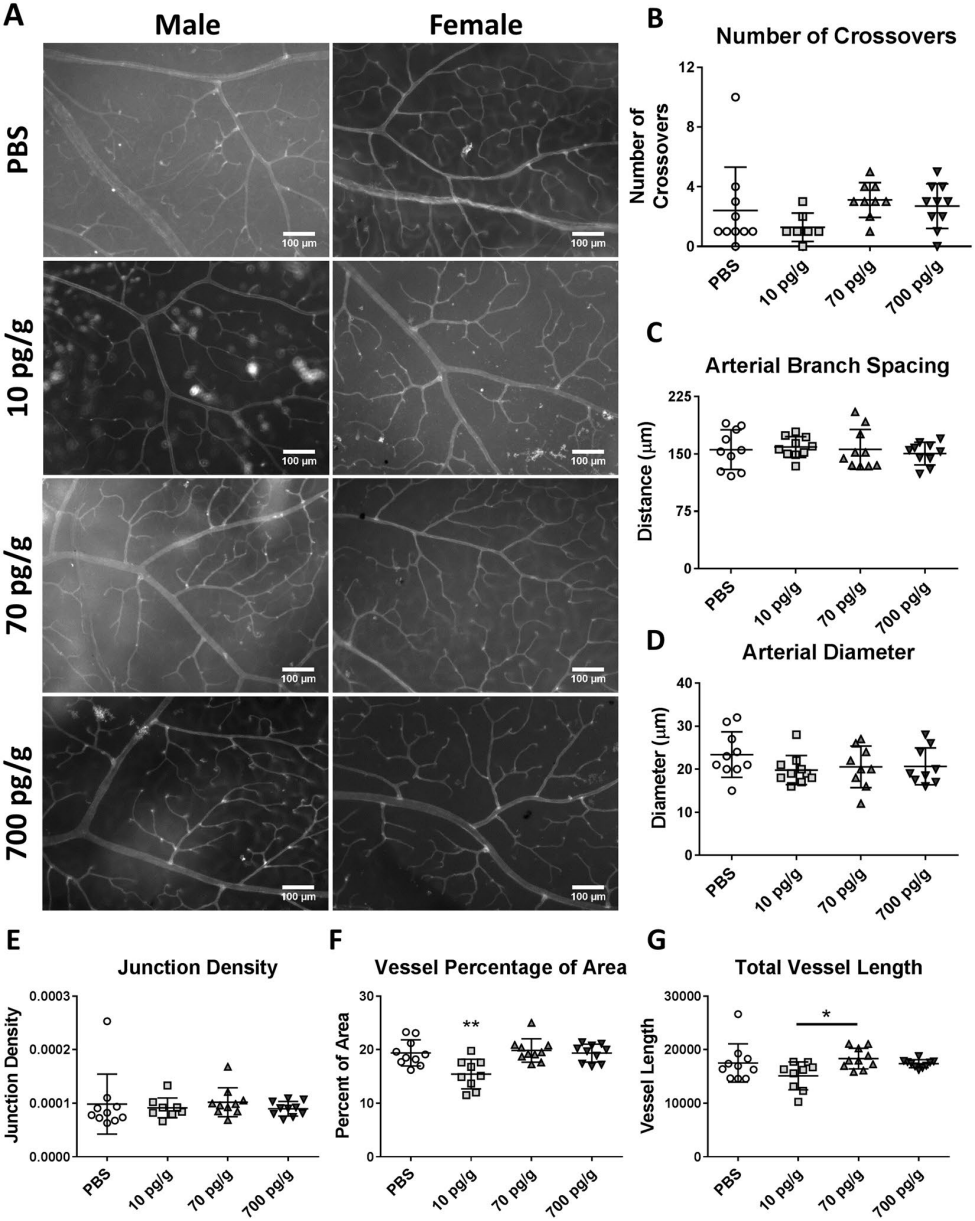
The retinal vasculature was compared between PBS-, 10 pg/g, 70 pg/g and 700 pg/g PGF-treated male and female mice (**A**). Vessels (white) were stained with TRITC-conjugated IB4. ImageJ and Angiotool were used for analysis. There was no significant difference in the number of crossovers (**B**), arterial branch spacing (**C**), arterial diameter (**D**) or junction density (**E**). Vessel percentage of the area was significantly lower in 10 pg/g PGF-treated mice compared to PBS-treated mice as well as 70 pg/g and 700 pg/g PGF-treated animals (significance compared to PBS-treated shown, **F**). Total vessel length was also less in 10 pg/g PGF-treated mice compared to 70 pg/g PGF-treated mice (**G**). Data was analyzed using one-way ANOVAs. Graphs show individual values with mean ± SD. * corresponds to p < 0.05 and ** corresponds to p < 0.01.

Representative images of the vascular plexus are presented in Fig. 8A. There was no difference in the number of arteriovenous crossovers (Fig. 8B), arterial branch spacing (Fig. 8C), arterial diameter (Fig. 8D), junction density (Fig. 8E), the number of abnormal veins, the distance from optic disc to plexus, arterial tortuosity, venous diameter, average vessel length or the total number of vessel end points (data not shown) between any of the groups. The percentage of area occupied by vessels was significantly less in the 10 pg/g PGF-treated group compared to PBS-treated (p = 0.0033), 70 pg/g PGF-treated (p = 0.001) and 700 pg/g PGF-treated (p = 0.0036) animals (Fig. 8F). When examined by sex, 10 pg/g PGF-treated males had significantly lower percentage of area occupied by vessels than PBS-treated (p = 0.0275), 70 pg/g PGF-treated (p = 0.0006) and 700 pg/g PGF-treated (p = 0.0153) males. 10 pg/g PGF-treated females tended to have lower percentage of area occupied by vessels but the difference was not statistically significant (data not shown). Similarly, 10 pg/g PGF-treated mice had lower total vessel length than 70 pg/g PGF-treated mice (p = 0.0324; Fig. 8G). Stratification by sex revealed 70 pg/g PGF-treated males had significantly higher total vessel length relative to PBS-treated (p = 0.0458) and 10 pg/g PGF-treated (p = 0.0003) males. 700 pg/g PGF-treated males also had significantly higher total vessel length that 10 pg/g PGF-treated males (p = 0.0327) but were not different from PBS-treated controls. There was no significant difference in total vessel length between doses in the female mice (data not shown). Although distance to the plexus and junction density had significant sex*dose interactions, there was no significant difference between groups on post-hoc testing. Overall, postnatal treatment with PGF did not have a robust effect on the developing retinal vasculature.

Smooth muscle coverage of the retinal arterioles was also investigated with α-actin staining. Although staining intensity tended to be lower in PBS and 10 pg/g PGF-treated female retinas, there was no qualitative difference between any treatment groups (Supplementary Fig. 2). Postnatal PGF treatment did not appear to affect retinal arteriole mural cell coverage.

## Discussion

PGF has been tested as a maternal treatment for PE in rodent and non-human primate models^54–56^. Although PGF reverses hypertension and renal histopathology pathognomonic of PE, it does not affect fetal or placental weights or fetal viability^54,55^. Since PGF is a 25 kDa glycoprotein that is thought to be too large to cross the placenta, administration in the postnatal period may have more therapeutic potential for offspring. In this study, mice were treated as pups from P1 to P10. As mice are weaned at P21, this represents a significant portion of the developmental period and corresponds to the preterm human brain of 28–30 weeks gestational age, highly relevant to preterm preeclampsia-affected infants. However, future work should also examine the effect of PGF-treatment in P12 and older mice as a model for the impact on the term human brain. Three doses were used to determine if a linear dose-response relationship existed and cerebrovasculature, neuroanatomy and behavior were examined in adult animals to determine if early postnatal treatment had a permanent effect. No dose response relationship was identified in this study, using two physiological and one supraphysiological doses.

Postnatal PGF treatment impacted behavior in adult *Pgf*^*−/−*^ mice. Depressive-like behaviour was lessened in 700 pg/g PGF treated female mice. Conversely, 10 pg/g PGF treatment appeared to negatively affect adult behavior, as anxiety-like behavior increased. This rise in anxiety-like behaviour may be linked to the demonstrated decreased in absolute volume of the amygdala in the 10 pg/g PGF-treated mice.

MRI volumetric assessment of PGF-treated mice found that brain structure volume was altered at 5–6 months by early postnatal treatment. However, the dose-response relationship was not linear. Instead, the 10 pg/g and 700 pg/g doses appeared to have little effect on brain structure volumes while the physiological peak maternal plasma dose of 70 pg/g had the most effect. Regarding specific structures, the 70 pg/g dose group exhibited larger volume of the occipital lobe cortex, inferior colliculus and medulla than PBS-treated *Pgf*^*−/−*^ mice. In a previous study that compared *Pgf*^*−/−*^ to congenic *Pgf*^*+/+*^ mice, *Pgf*^*−/−*^ mice had smaller volumes of the occipital lobe, inferior colliculus, medulla, arbor vitae of cerebellum, cerebellar cortex, inferior cerebellar peduncle, entorhinal cortex, parieto-temporal lobe, olfactory bulbs, and pre-parasubiculum and displayed deficits in spatial memory (Y-maze performance)^26^. Since no deficit in spatial memory was found in the current study, smaller size of the parieto-temporal lobe and entorhinal cortex may underlie this phenotype.

Examination of the cerebrovasculature by µCT imaging in adult treated animals revealed no difference in the number or diameter of vessels segments. Likewise, there was no difference in the adult retinal vasculature after early postnatal PGF treatment, even though the retina vascularizes during the P1–10 treatment period. These results contrast with those of other studies that have examined PGF treatment in models of myocardial infarction and ischemic stroke that demonstrated improvements in vascularization^28–35^. Overall, early postnatal treatment of *Pgf*^*−/−*^ with PGF had an impact on adult neuroanatomy and behaviour rather than on the cerebrovasculature. This could represent greater responsiveness of the neural tissue to PGF. Alternatively, the bioavailability of the systemically administered PGF may have been too low to affect the cerebrovasculature or physiological pro-angiogenic cues may have obscured angiogenesis promoted by the injected PGF. Further, alterations to the vasculature during the postnatal period may not have persisted into adulthood when the vessels were assessed. As postnatal treatment is temporally offset from the developmental insult, it may not be capable of fully reversing PE-induced changes. Although the cerebrovasculature is understudied in this area, in one report, stabilization of hypoxia-inducible factor (HIF)-1α upregulated hippocampal VEGF and improved episodic memory in mice^57^. In rats, upregulated brain VEGF expression in the early postnatal period increases brain vascularity as well as the number of neurons, astrocytes, oligodendrocytes and neural stem cells^58^. Previously, Carver *et al*. demonstrated reversal of the detrimental developmental effects of maternal PE phenotype on behavior and neuroanatomy in a mouse model after prenatal pravastatin treatment^59,60^. Pravastatin has been linked to upregulation of PGF in the placenta^61^, suggesting that increased PGF during the appropriate developmental period is capable of protecting the offspring from PE. Optimization of the postnatal dose and appropriate choice of pro-angiogenic factor may still provide benefit.

Use of a different angiogenic/neurotrophic factor may be more effective at correcting PGF deficiency or PE-related deficits. Fibroblast growth factor is protective against histological damage in rat models of neonatal hypoxic-ischemic brain injury^62^. Other interventions including folic acid and erythropoietin are protective against behavioral and neuroanatomical changes after traumatic brain injury in piglet and rat models^63,64^. A cocktail of factors may also be more effective. PGF treatment may be more effective in the context of perinatal complications other than PE. For example, asphyxiation and perinatal stroke are more similar to ischemic diseases like myocardial infarction and stroke, both of which have benefited from PGF treatment in animal models^28–30,33,34^.

Non-pharmacological interventions are also possible and should be considered. The human brain is not fully developed at birth but continues to develop through childhood with cognition and brain structure impacted by individual experiences. In children, insults like early life stress have been linked to impaired performance on a spatial working memory tasks and smaller prefrontal cortex volume^65^. Conversely, training interventions can improve cognition and alter brain resting state connectivity^66^. Mice appear to respond similarly to insults and enrichment. Exposure to an impoverished environment beginning at 3 weeks of age in mice weakened spatial learning while an enriched environment enhanced spatial learning^67^. An enriched environment also rescues spatial learning in mice exposed to prenatal stress^68,69^.

## Conclusions

In *Pgf*^*−/−*^ mice, postnatal treatment with PGF altered the adult neuroanatomical and behavioral phenotype. These results suggest that asynchronous neonatal treatment of developmental alterations in the brain associated with PGF deficiency may be possible. In the future, these results may have relevance to infants that experienced low PGF adverse prenatal conditions. However, clinical translation of postnatal therapies to ameliorate developmental insults will depend on clarification of the PE-F1 phenotype. Furthermore, the relative value and risks of a pharmacological intervention compared to environmental and educational enrichment must be established.

## Supplementary information

Supplementary Information
