# Supplementary material for: Adult Pgf−/− mice behaviour and neuroanatomy are altered by neonatal treatment with recombinant placental growth factor
Source: Sci Rep. 2019 Jun 26;9:9285. doi: 10.1038/s41598-019-45824-6 (PMC6594955; doi:10.1038/s41598-019-45824-6)
Supplement: Supplementary file 1 — Supplementary Information [file 41598_2019_45824_MOESM1_ESM.pdf]

Adult *Pgf*$^{-/-}$ mice behaviour and neuroanatomy are altered by neonatal treatment with recombinant placental growth factor.

Vanessa R. Kay[a*], Lindsay S. Cahill[b], Anas Hanif[a], John G. Sled[b,c], Peter Carmeliet[d],

Chandrakant Tayade[a], B. Anne Croy[a]

[a] Department of Biomedical and Molecular Sciences, Queen's University, Kingston, ON, Canada, K7L 3N6

[b] Mouse Imaging Centre, Hospital for Sick Children, Toronto, ON, Canada, M5T 3H7

[c] Department of Medical Biophysics, University of Toronto, ON, Canada, M5T 3H7

[d] Laboratory of Angiogenesis and Vascular Metabolism, VIB - Vesalius Research Center, University of Leuven, Department of Oncology, Leuven Belgium

***Corresponding Author:**

Vanessa R. Kay

8vrk@queensu.ca

613-533-2600 x74917

Rm 823 Botterell Hall

Queen's University

Kingston, ON

Canada

Supplementary Figure 1:

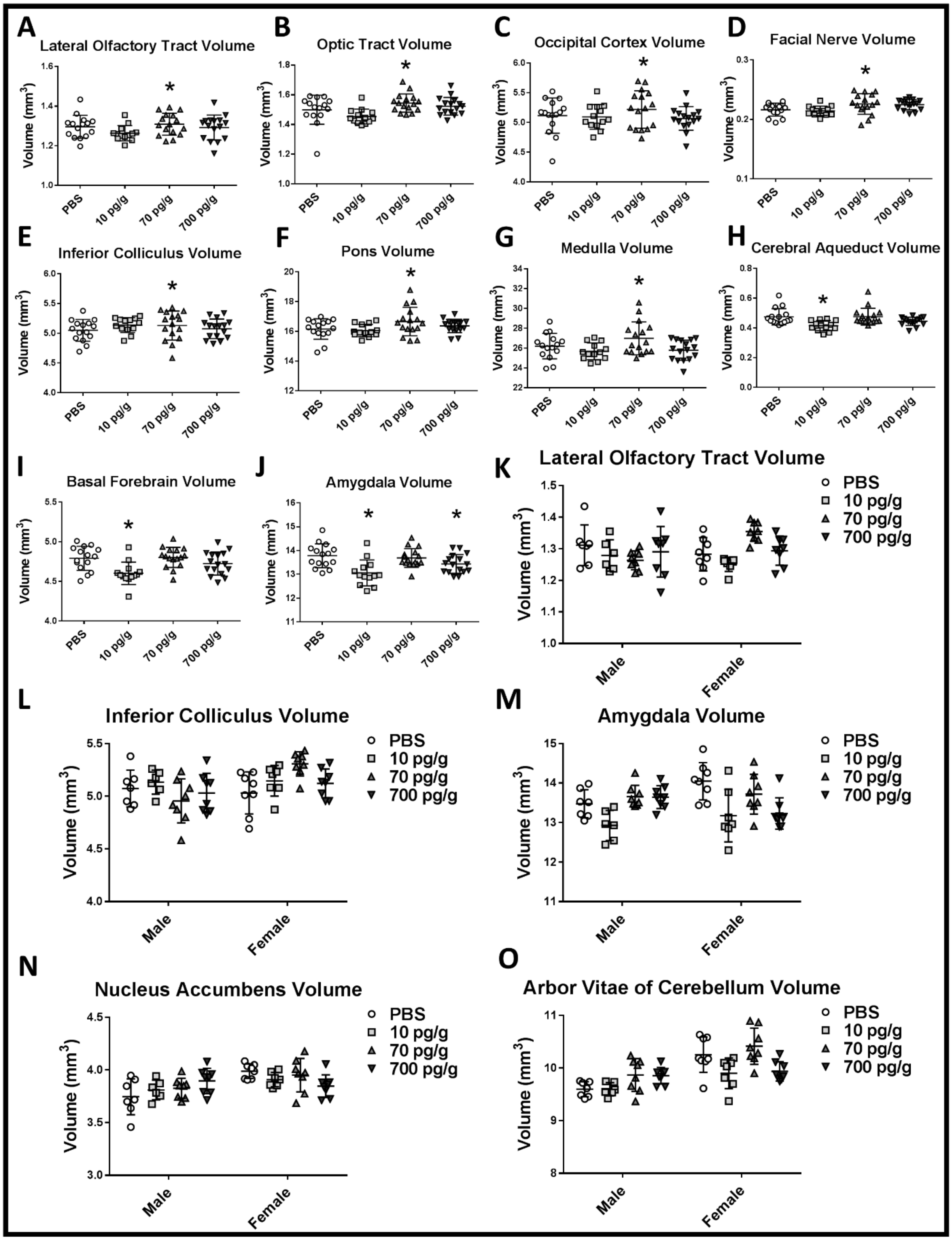

Supplementary Figure 1: Dose response relationships were examined in brain structures that differed significantly between groups (A-J). In the cerebral aqueduct and basal forebrain (A,F), volumes were significantly smaller only in the 10 pg/g PGF-treated group. Likewise, amygdala volume (E) was significantly less in the 10 pg/g and 700 pg/g PGF-treated groups. Volumes of the lateral olfactory tract (B), optic tract (C), occipital cortex (D), facial nerve (G), inferior colliculus (H), pons (I) and medulla (J) were significantly greater in the 70 pg/g PGF-treated group but similar to PBS-treated control in the 10 pg/g and 700 pg/g PGF-treated groups. Significant sex-dose interactions were present in the lateral olfactory tract (K), inferior colliculus volume (L), amygdala (M), nucleus accumbens (N) and arbor vitae of the cerebellum (O). There were 16 mice per treatment group with 4 excluded for poor perfusion quality. Graphs show individual values with mean ±SD. White circles represent PBS-treated mice, light grey squares represent 10 pg/g PGF-treated mice, medium grey triangles represent 70 pg/g PGF-treated mice and dark grey inverted triangles represent 700 pg/g PGF-treated mice. * corresponds to FDR < 0.1 compared to the PBS-treated group.

Supplementary Figure 2:

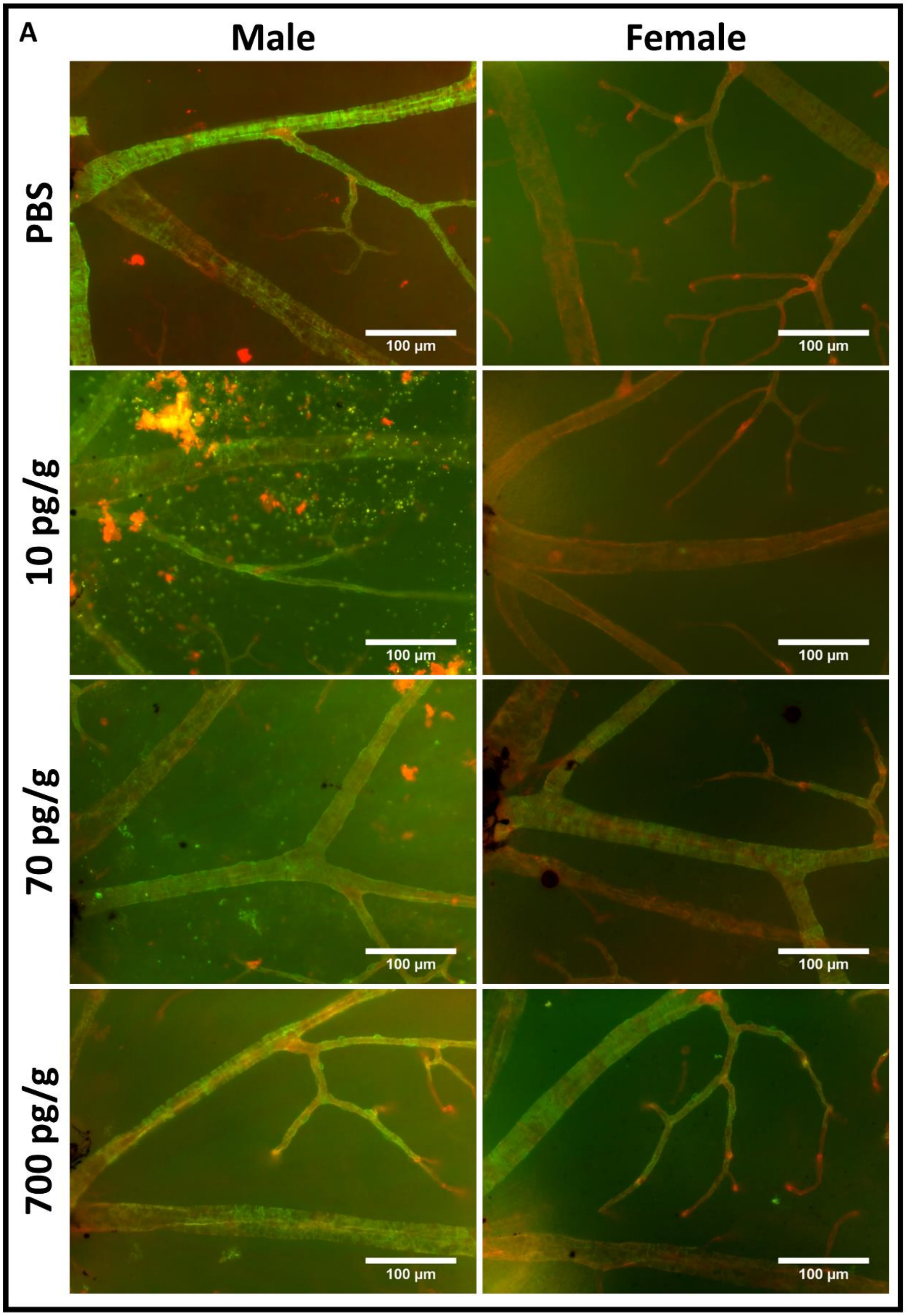

Supplementary Figure 2: Smooth muscle cell coverage of retinal vessels after postnatal PGF treatment. The mural cell coverage of the retinal arterioles and venules was assessed using TRITC-conjugated IB4 to identify vessels (red) and Alexa Fluor 488-conjugated anti α-actin to identify smooth muscle (green). Staining intensity was qualitatively less in PBS- and 10 pg/g PGF-treated females but there was no apparent difference in SMC coverage between any doses (A).
